# AutoAnnotate: A Cytoscape app for summarizing networks with semantic annotations

**DOI:** 10.12688/f1000research.9090.1

**Published:** 2016-07-15

**Authors:** Mike Kucera, Ruth Isserlin, Arkady Arkhangorodsky, Gary D. Bader

**Affiliations:** 1 The Donnelly Centre, University of Toronto, Toronto, Canada

**Keywords:** network analysis, enrichment map, tag cloud, network clustering, complexity reduction, modular networks, annotations, cytoscape

## Abstract

Networks often contain regions of tightly connected nodes, or clusters, that highlight their shared relationships. An effective way to create a visual summary of a network is to identify clusters and annotate them with an enclosing shape and a summarizing label. Cytoscape provides the ability to annotate a network with shapes and labels, however these annotations must be created manually one at a time, which can be a laborious process. AutoAnnotate is a Cytoscape 3 App that automates the process of identifying clusters and visually annotating them. It greatly reduces the time and effort required to fully annotate clusters in a network, and provides freedom to experiment with different strategies for identifying and labelling clusters. Many customization options are available that enable the user to refine the generated annotations as required. Annotated clusters may be collapsed into single nodes using the Cytoscape groups feature, which helps simplify a network by making its overall structure more visible. AutoAnnotate is applicable to any type of network, including enrichment maps, protein-protein interactions, pathways, or social networks.

## Introduction

Identifying clusters of nodes in a network, based on similarity of node attributes or connectivity between the nodes, is useful for defining groups of related nodes. For example, clusters in a protein-protein interaction network often represent molecular complexes, proteins that work together as a group to perform a specific function ^1^, whereas clusters in a co-authorship network represent a group of authors that often collaborate and publish together.

**Figure 1.**
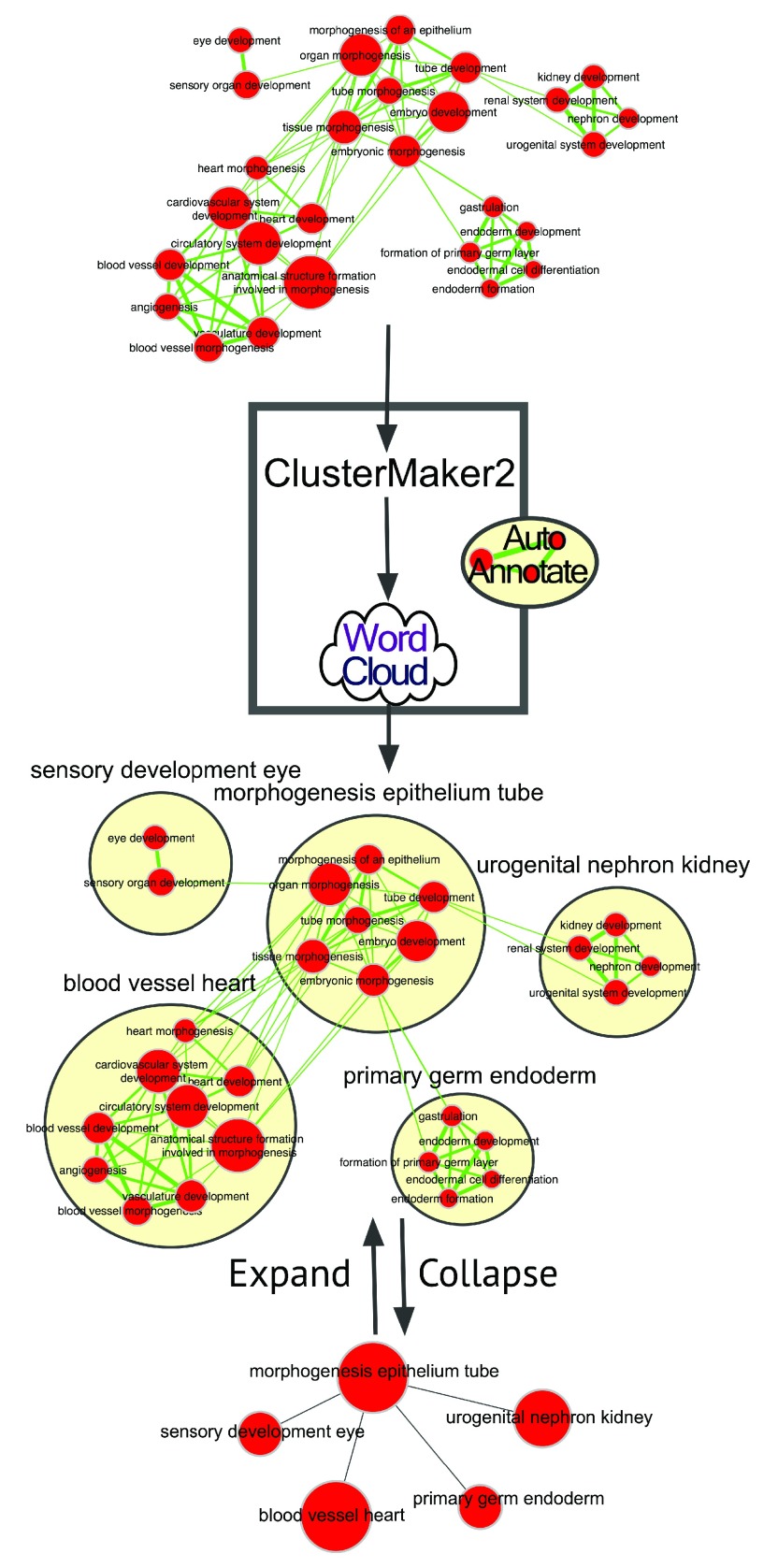
AutoAnnotate overview. A network is clustered, textual annotation associated with each cluster is automatically summarized as a single cluster label, and the results are visualized. Users can customize the view by selectively collapsing and expanding clusters.

Clusters can be used to create a visual summary of a network by drawing an enclosing shape around each cluster and adding a textual summary label next to each cluster ( Figure 1). This technique is often effective in summarizing the results of a network analysis by highlighting main themes and categories within the network. AutoAnnotate ( http://apps.cytoscape.org/apps/autoannotate) was originally created to aid pathway enrichment analysis using the Enrichment Map App ^2^ as every enrichment map requires clustering and annotation, but AutoAnnotate is now being made available as a stand-alone app to benefit other types of analysis.

Cytoscape enables users to add annotations on top of the network, including arrow, image, shape and text objects. However, fully annotating the clusters in a network can be cumbersome because the user must manually create and position each individual annotation. Furthermore, Cytoscape does not maintain any relationship between the network layout and the annotations, when the layout changes the user must manually reposition the annotations. Because of these limitations some users prefer to export their networks and add annotations using an external application, such as Adobe Illustrator.

AutoAnnotate is a Cytoscape 3 App that identifies clusters and automatically draws shape and label annotations for each cluster. The enclosing shapes make it visually clear which nodes belong to each cluster when the clusters do not overlap. The generated labels provide a concise semantic summary of the data attached to the nodes in each cluster. AutoAnnotate maintains a relationship between the annotations and the nodes in a cluster, if the layout of the network changes then the annotations are automatically repositioned. AutoAnnotate maintains multiple sets of annotations for a single network, which allows the user to experiment with different clustering algorithms and label generation strategies. Additionally, AutoAnnotate allows clusters to be collapsed, which can simplify large networks by reducing potentially large sections of the network into single nodes.

AutoAnnotate leverages two existing Cytoscape Apps to do most of its work: clusterMaker2 ( http://apps.cytoscape.org/apps/clustermaker2) and WordCloud ( http://apps.cytoscape.org/apps/wordcloud). The clusterMaker2 App provides several clustering algorithms and is directly called by AutoAnnotate to identify clusters of nodes in the network. The use of clusterMaker2 is optional, and the user may provide their own list of cluster identifiers. WordCloud is a Cytoscape App that creates a visual summary of selected attributes for a set of nodes by displaying a word tag cloud, where more frequent words are displayed using larger font size and adjacent words are grouped closer together. AutoAnnotate invokes WordCloud to generate a word tag cloud for the node data within each cluster, which is used to derive the text for the label annotations.

AutoAnnotate can be installed from within Cytoscape via the App Manager or from the Cytoscape App Store.

## Operation

This section discusses a basic overview of the capabilities of AutoAnnotate. A more detailed user manual is available from http://baderlab.org/Software/AutoAnnotate.

AutoAnnotate creates and manages a list of “Annotation Sets” for each network. An Annotation Set consists of a group of clusters and their associated annotations. Each network may have one active Annotation Set at time. The user may create as many Annotation Sets as they like, and can easily switch between them. The use-case for supporting multiple Annotation Sets is to allow the user to experiment with different clustering and summarization parameters, and then choose the most satisfactory resulting Annotation Set. Annotation Sets are saved to the Cytoscape session file.

**Figure 2.**
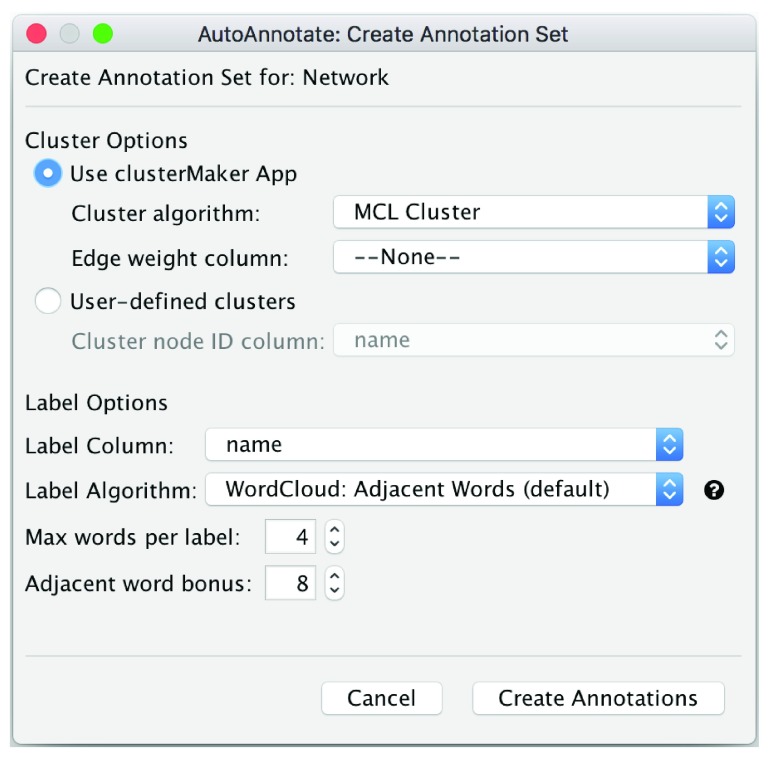
The Create Annotation Set dialog. Cluster options: ClusterMaker2 requires a clustering algorithm and edge weight attribute to be selected, otherwise a node attribute must be selected. Label options: User selects the node attribute to use for creating labels, the labelling algorithm to use, and the maximum number of words per label.

To create an Annotation Set select **AutoAnnotate > New Annotation Set…** from the **Apps** menu. A dialog will pop up enabling the users to define the clusters in the network ( Figure 2). Clusters can be defined in two ways: user defined or calculated automatically through AutoAnnotate. AutoAnnotate accepts any node attribute associated with the network to define clusters. Nodes with the same value for the attribute will be placed in the same cluster. This enables users to import cluster definitions from external programs or clustering algorithms. Alternately, clusters can be calculated within Cytoscape using the clusterMaker2 App ^3^ which enables users to cluster a network using one of many clustering algorithms it provides. The clusterMaker2 algorithms are available as commands or from the Apps menu. Most of the algorithms can generate a cluster ID attribute in the node table, which can be consumed by AutoAnnotate. For convenience, AutoAnnotate also provides access to a subset of the clusterMaker2 algorithms directly from the Create Annotate Set dialog ( Figure 2). This enables a user to quickly get started using AutoAnnotate without knowing the fine details of clusterMaker2. Currently, due to the simplified interface within AutoAnnotate, only clustering algorithms that take zero or one parameters and that run quickly are available directly from AutoAnnotate. After the clusters are computed, a label for each cluster is generated by the WordCloud app ^4^.

**Figure 3.**
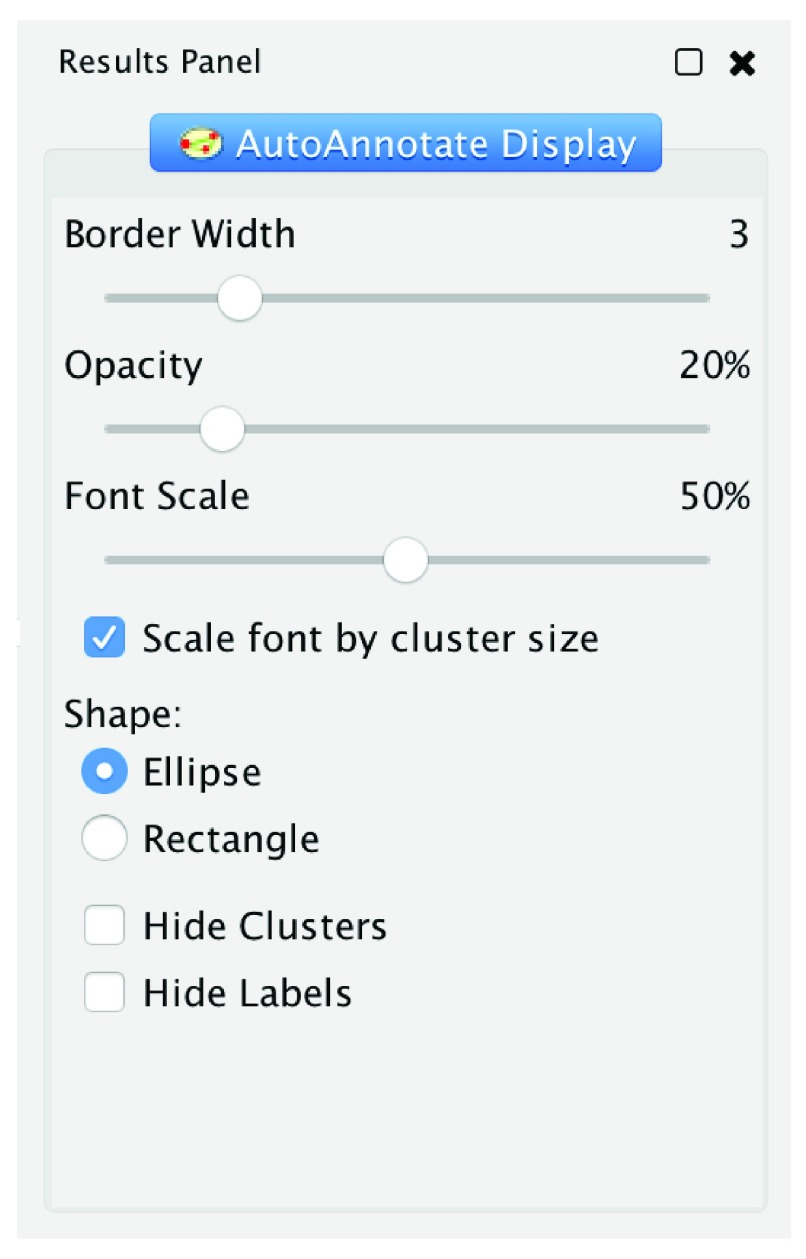
The Display Options panel. Changing parameters here automatically updates the display.

Once clusters and labels are calculated, AutoAnnotate automatically adds them as annotations to the network ( *e.g*. ellipses around each cluster). Clusters are contained within bounding annotation shapes with its corresponding label as a text annotation directly above it. The look of the label and shape annotations may be modified using the Display Options panel ( Figure 3). From this panel the user can adjust border width, shape type, opacity and visibility for shape annotations, and font size, font scaling and visibility for label annotations.

**Figure 4.**
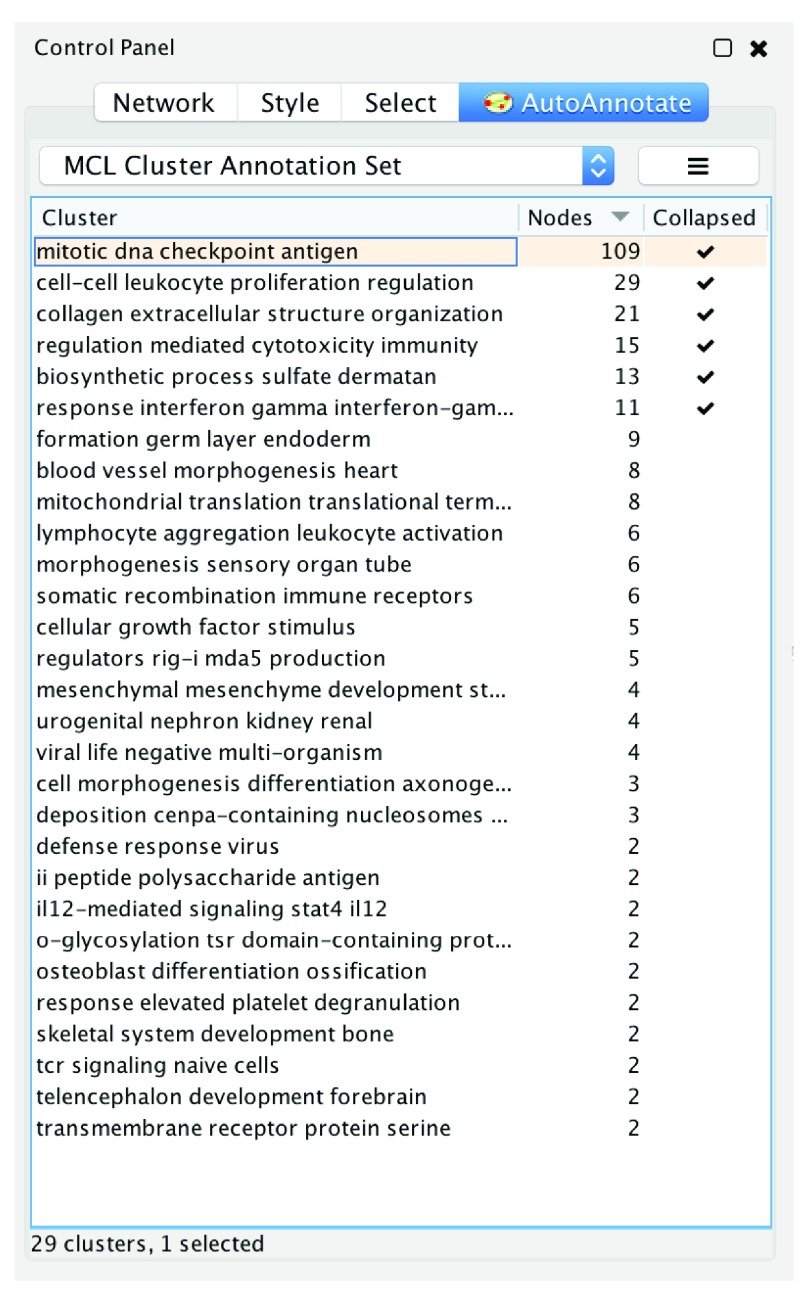
The main Annotation Set panel. Clusters and labels are shown. Clusters can be collapsed or expanded. An annotation can be customized via options available in a context sensitive (right click) menu.

After an Annotation Set is created, it is shown in the main AutoAnnotate panel ( Figure 4). This panel allows the user to choose which Annotation Set is currently active for each network. When an Annotation Set is active, a list of all its clusters and labels is displayed in the table. Each row represents one annotation ( *i.e*. one cluster with its computed label) with the number of nodes it contains and whether it is collapsed or not.

Although AutoAnnotate aims to automatically create the best labels and annotations, it also enables users to customize the resulting annotations. Clusters and labels can be manually adjusted. Multiple clusters can be combined. New clusters can be created from selected nodes directly in the network view. Labels can be re-generated automatically based on different label options. New Annotation Sets can be created by selecting a subset of clusters from an existing Annotation Set.

To further simplify complex networks AutoAnnotate has the ability to collapse some or all of the clusters automatically. The set of nodes belonging to a cluster are removed and replaced with a single group node representing the set. The group node is named using the computed label. When all clusters in a complex network are collapsed, it can substantially decrease the complexity of the network so that major themes or structure of the underlying network is more readily apparent.

## Implementation

### Feature implementation

WordCloud registers a command service that calculates a word-cloud given a set of nodes and one or more node attributes. AutoAnnotate calls this command once for each cluster. The results of the command are placed in a list of WordInfo objects, which contain the font size of each word, the order that words appeared in the attributes (maintains word order), and the word adjacency group that contains the word. The word adjacency group is an identifier assigned to each word based on the number of times words occur in adjacent positions.

WordCloud computes word frequencies which AutoAnnotate then uses to calculate the best combination of words to describe the given cluster, considering word frequency in the cluster compared to that in the entire network. Normally WordCloud returns all of the words in the cloud, however AutoAnnotate uses a maximum of 1–10 words to make a label, and therefore must make a decision of which words from the cloud to use and in which order. AutoAnnotate currently has two options for deciding this. The “Biggest Words” option sorts the words by font size, takes the N largest words, then sorts the result by word order (this preserves the original order that the words appeared in the selected attribute). The “Adjacent Words” option is a heuristic that attempts to balance word size with word adjacency information. First the words are sorted by font size, then a size bonus is added to every word that is in the same adjacency group as the N largest words. This causes words that are in the same group as the N largest words to be more likely to be chosen. The size bonus cannot cause a word to become bigger than the largest word in the group. Then the list is sorted again by size and the N largest words are selected. We have found through trial and error with many networks that a size bonus of eight results in labels that provide a good semantic description of the nodes in the cluster. Thus, we have made this the default label making option.

The Cytoscape group nodes feature is used to collapse clusters. When AutoAnnotate collapses a cluster it first creates a group node that contains all the nodes in the cluster and then the group node is collapsed. The shape and label annotations are no longer drawn for the collapsed cluster. When the cluster is collapsed Cytoscape will create "meta-edges" between the group node and any other nodes it is connected to. The collapsed group nodes and the meta-edges provide a summary of the network. When a cluster is expanded the group node is first expanded and is then deleted. Collapsed groups must be expanded before switching to another Annotation Set because the nodes contained in each group may belong to different clusters in the other Annotation Set.

### Software design

AutoAnnotate is a Cytoscape bundle App based on the Cytoscape 3-supported OSGi (Open Services Gateway Initiative) ( https://www.osgi.org/developer/specifications/) module framework. AutoAnnotate version 1.1 depends on Java 8, the Cytoscape 3.3 API and WordCloud 3.1. The Cytoscape App Manager or the Cytoscape App Store installs WordCloud automatically when installing AutoAnnotate. ClusterMaker2 is not installed automatically because it is optional. To make installation of clusterMaker2 easier for the user the Create Annotation Set dialog attempts to detect the presence of clusterMaker2, and if not available the user is presented with a web link to the App Store page for clusterMaker2 from which they can install it.

Cytoscape makes its extensive API available to apps via a number of OSGi service interfaces. These services must be acquired in a bundle activator lifecycle method that is called when the App is initialized. Traditionally the service references are passed down the chain of constructors that build the App’s object graph. This technique is known as Dependency Injection, since a class’ dependencies are “injected” when the class is instantiated rather than the class having the responsibility of looking up the dependencies it needs. Dependency Injection is a well known software design pattern that improves modularity and facilitates unit testing. However, in Cytoscape this Dependency Injection is traditionally done manually, by hand-coding constructor parameter lists, which is verbose and error prone. AutoAnnotate uses the Google Guice Dependency Injection framework ( https://github.com/google/guice/wiki/GettingStarted) along with the Peaberry OSGi adapter ( https://github.com/google/guice/wiki/OSGi). Guice is configured to acquire Cytoscape services through Peaberry, and those services are then made available to all objects instantiated through Guice, removing the need to hand code constructor method parameter lists. The result is cleaner code that is easier to maintain and unit test.

The internal architecture of AutoAnnotate is divided into three main modules and some smaller supporting modules. The three main modules are: 1) Data Model, which is controlled by the Model Manager and contains the model for Annotation Sets, clusters, display options and labels. The Data Model is saved to the Cytoscape session file. 2) The UI, which consists of the main panel ( Figure 4), the display options panel ( Figure 3), the create annotation set dialog ( Figure 2), menu items, and various other information and warning dialogs. 3) The Annotation Renderer, which draws the label and shape annotations on the network canvas.

**Figure 5.**
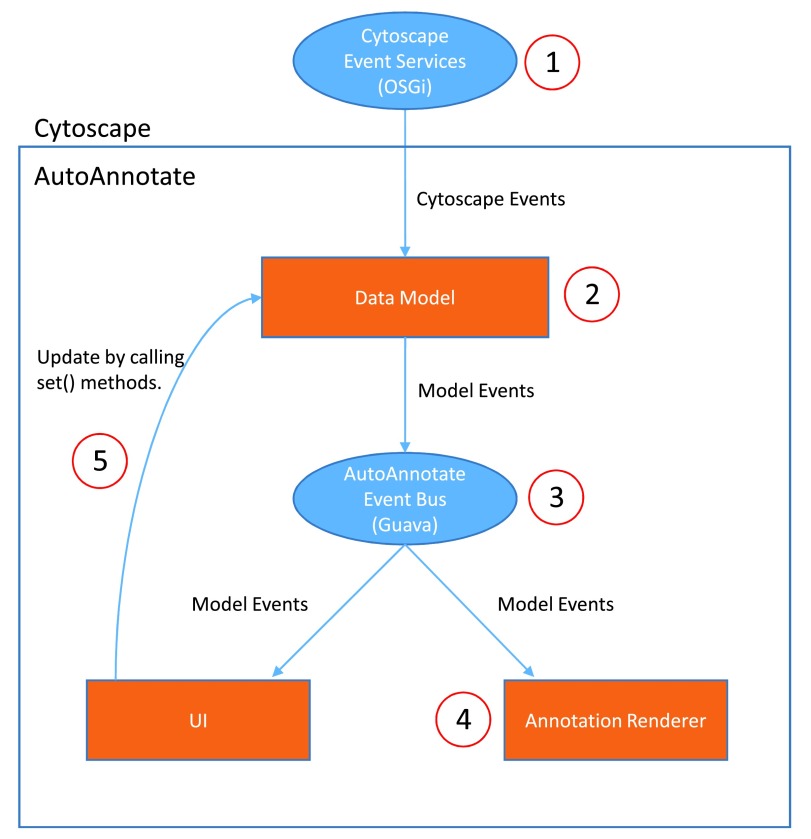
Event Flow. 1) Cytoscape events are fired using OSGi services ( *e.g.* SetCurrentNetworkViewEvent, NetworkViewAboutToBeDestroyedEvent). 2) The AutoAnnotate Model Manager reacts to Cytoscape events and updates the Data Model. 3) The ModelManager fires Model Events over the Guava EventBus ( *e.g.* AnnotationSetAddedEvent, ClusterAddedEvent). 4) The UI and Annotation Renderer modules each respond to a subset of the model events. There is no direct dependency between the UI and the Annotation Renderer. 5) The UI directly updates the Model, which causes Model Events to fire, which updates the UI and Annotations.

The Model Manager listens to Cytoscape events, such as events for nodes being selected, and modifies the Data Model to stay in sync with Cytoscape. The Model Manager emits Model Events whenever the Data Model changes, caused by reacting to Cytoscape events or caused by user interaction. Client modules that listen to model events do not use the traditional observer pattern ^5^, which is ubiquitous in object oriented user interface programming, instead the EventBus class from the Google Guava library is used. An EventBus allows event subscribers to be fully decoupled from event sources since each source does not need to maintain a listener list. The Model Manager emits the Model Events over the EventBus and the other main modules (UI and Renderer) subscribe to the EventBus and declare which event types they want to receive. Subscribing to the EventBus is very simple since it is injected by Guice. When the user interacts with a control in one of the UI panels a mutator method is called on the data model, this in turn fires a model event, which causes the rest of the UI to be updated. The UI and network view are kept in sync with the model without introducing any direct dependencies between the UI panels, the annotation renderer or the data model. Figure 5 depicts the event flow.

Integration with the WordCloud and clusterMaker2 Apps is made possible via the Cytoscape command-executor API ( http://chianti.ucsd.edu/cytoscape-3.4.0/API/org/cytoscape/command/package-summary.html). ClusterMaker2 registers a command service for each of the clustering algorithms it provides. AutoAnnotate calls these commands using the CommandExecutorTaskFactory service.

## Results and discussion

We present four use cases for AutoAnnotate. The first, the most common use case, is an enrichment map. Clusters in an enrichment map represent similar pathways and processes. AutoAnnotate offers a way to easily summarize the main themes present in the network and is used routinely by enrichment map users. The second use case further demonstrates AutoAnnotate summarization capabilities by demonstrating how an annotated network can be collapsed to just its major themes. The third use case demonstrates how AutoAnnotate can be used for a different type of network, a co-authorship network. Finally, the fourth use case demonstrates another network type, a protein-protein interaction (PPI) network annotated with Gene Ontology (GO) terms ^6^, to demonstrate how multiple annotation sets for an individual network can be used to summarize the same network to get different results.

## Use case 1 - Enrichment maps

**Figure 6.**
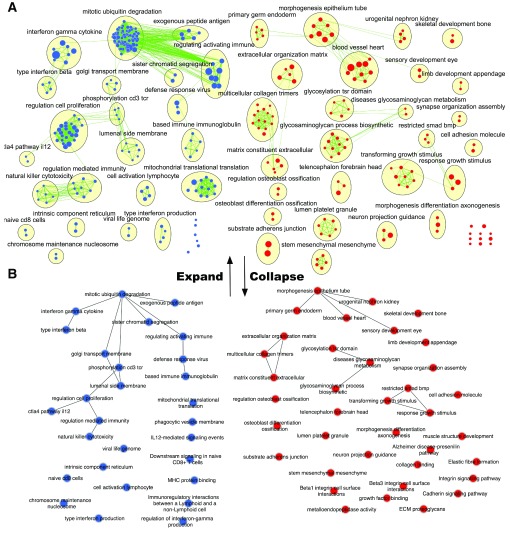
AutoAnnotated enrichment map. ( **A**) Annotated enrichment map ( **B**) Collapsed enrichment map.

An enrichment map is a graphical representation of a pathway enrichment analysis where nodes represent pathways and edges the crosstalk (or shared genes) between connecting pathways. To illustrate this, we downloaded gene expression from the TCGA Ovarian serous cystadenocarcinoma RNASeq V2 cohort on 2015-05-22 from cBioPortal for Cancer Genomics ( http://www.cbioportal.org/data_sets.jsp). Enrichment analysis was conducted on a subset of this data as described in the enrichment map protocol ^7^. As an example, the resulting enrichment map was annotated using AutoAnnotate ( Figure 6A).

Enrichment results from an ovarian cancer gene expression analysis are depicted as an enrichment map (q-value<0.0001, enrichment analysis performed as per EM protocol ^7^). The resulting enrichment map was annotated using parameters - WordCloud normalization of 0.5, MCL clustering, and pathway description was used for label calculation ( Figure 6A). WordCloud calculates the word frequencies of the attribute selected. For an enrichment map we chose the pathway description attribute to calculate labels as it contains a concise description of each node. The normalization factor controls how the word frequencies are weighted compared to the words in the rest of network. With a normalization factor of zero the significance of each word is calculated solely on how many occurrences it has in the given cluster. This may cause very frequent words within the network such as “pathway” or “regulation” to be prominent in annotations as they are found often in pathway descriptions. By increasing the normalization factor, we increase the weight calculated from the ratio of a word frequency in the cluster to its frequency in the entire network to diminish the presence of these recurrent words in the cluster labels.

## Use case 2 - Collapsed AutoAnnotated enrichment map

In an enrichment map visualization (and networks of other types) our gaze often gravitates towards the largest most densely connected region or cluster. In an enrichment map such clusters do not necessarily represent the most important themes of the analysis but instead biological pathways that are well studied and documented in public databases. AutoAnnotate allows users to normalize this bias by collapsing the network. Both large and small clusters become a single node labelled with the computed annotation and nodes not part of any cluster retain their original pathway or node name. In this format large clusters are no more important than single nodes but it is easy to quickly see the major themes of an analysis even within large complicated networks. The enrichment map was further summarized by collapsing all clusters to individual group nodes that represent the cluster and are named according to the annotation given. To Collapse the network simply select “Collapse All” from the AutoAnnotate Input panel menu ( Figure 6B).

## Use case 3 - Co-authorship network

**Figure 7.**
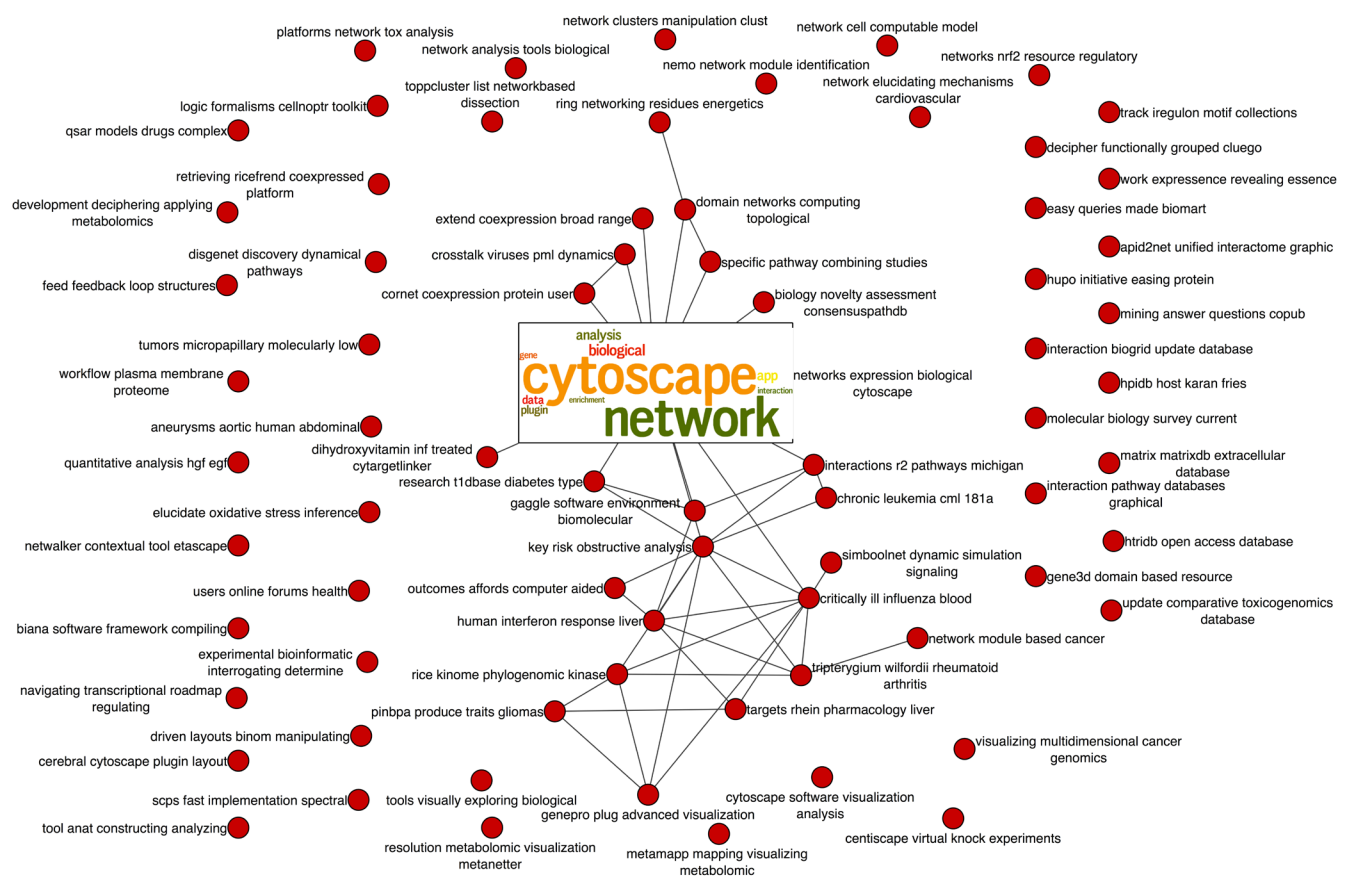
Co-authorship network for article containing ‘Cytoscape’ in its title/abstract/keyword. The most central node was annotated with an additional image representing the top words in the set of publications as generated by wordle.com.

AutoAnnotate is not limited to enrichment maps. Any standard network can be annotated based on node attributes in the network. For example, co-authorship networks where nodes are authors and edges connect two authors that have published together can also be annotated by AutoAnnotate. Using all the publications that had Cytoscape in its title/abstract/keyword as extracted from Scopus ^8^ using the SocialNetworkApp ^9^ we constructed a co-authorship network. The large network was filtered to contain only authors that had more than 20 citations. The resulting network ( Figure 7) was annotated using the ‘publications’ attribute that contains the titles of every article an author published (in this case any article that contained the word ‘cytoscape’ in its title/abstract/keywords for the given author). The WordCloud normalization factor was set to 0.3 (slightly smaller than the previous enrichment map use case). Given that the set of articles dealt exclusively with Cytoscape they shared many similar words and themes. A high normalization factor would have eliminated most of these similar words like ‘network’, ‘analysis’ and ‘biological’. We wanted to highlight the different areas that use Cytoscape as well as the prominent purposes. Experimenting with the WordCloud normalization factor allowed us to find the ideal balance. The annotated network was collapsed to show the varied themes of the articles relating to Cytoscape.

## Use case 4 - PPI network

**Figure 8.**
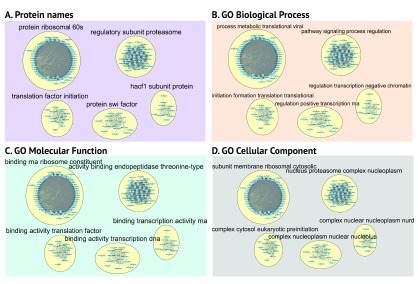
Running AutoAnnotate on a PPI network. Four different annotations for the same base PPI network using parameters - WordCloud normalization of 0, previously created cluster designations, and **A**. protein names, **B**. GO biological process, **C**. GO molecular function, or **D**. GO cellular component was used for label calculation.

AutoAnnotate enables the user to create multiple annotations based on different parameters and easily toggle between the different annotations. Figure 8 shows the annotation of a PPI network generated from co-purified complexes ^10^. A subset of complexes was annotated using different attributes including protein names, GO biological process, molecular function or cellular component. Depending on the attribute used to compute annotations the summarization gives a slightly different view of the data.

## Conclusion

AutoAnnotate aids in network analysis and interpretation by providing a concise visual summary of clusters in a network. Clusters can be visualized either as shape and text annotations overlaid on top of the network, or by collapsing the clusters into single nodes.

Users can get started quickly with AutoAnnotate by accepting the defaults when creating an Annotation Set. More advanced users can experiment with different strategies for generating clusters and labels. Users are free to specify clusters themselves manually or using other clustering tools.

AutoAnnotate fits into a larger workflow to help analyze a network or help present the results of an analysis. For example AutoAnnotate is an important part of the enrichment map protocol.

## Software availability

Homepage: http://baderlab.org/Software/AutoAnnotate

Software available from: http://apps.cytoscape.org/apps/autoannotate

Latest source code: https://github.com/BaderLab/AutoAnnotateApp

Archived source code as at the time of publication:

https://zenodo.org/record/57021#.V3vOgpMrJE4 ^11^

License: Lesser GNU Public License 2.1: https://www.gnu.org/licenses/old-licenses/lgpl-2.1.html
