# Supplementary material for: AutoAnnotate: A Cytoscape app for summarizing networks with semantic annotations
Source: F1000Res. 2016 Jul 15;5:1717. [Version 1] doi: 10.12688/f1000research.9090.1 (PMC5082607; doi:10.12688/f1000research.9090.1)
Supplement: Peer review history

# Peer review: Referee response for version 1

**DOI:** 10.5256/f1000research.9783.r16577

**Published:** 2016-10-24

**Authors:** Harm Nijveen

**Affiliations:** 1 Bioinformatics Group, Department of Plant Sciences, Wageningen University, Wageningen, Netherlands

AutoAnnotate is a Cytoscape app that can make complex networks easier to interpret by clustering nodes and labelling the clusters with relevant terms. Clusters can be collapsed into single nodes, greatly simplifying the network. The app is easy to use and allows configuration of quite of number of settings. The clusters are labelled based on the most frequent words in a (configurable) node column, using functionality from the WordCloud app.

 The proper labelling of the clusters can require some toying with the parameters and manual curation, both in AutoAnnotate and WordCloud. For instance, using the AlzheimerEM.cys network from the WordCloud tutorial a cluster was labelled with the terms “interphase cycle”, where “cell cycle” would be more appropriate. The word “cell” was omitted due to the default “Normalize” setting of the WordCloud app that prevented very frequent words to be present in a label. Lowering this setting from 0.5 to 0.3 changed the label to “cell cycle”. This is described in the quite elaborate AutoAnnotated User Guide.

 I have also tested the app with the SeedNet network (http://netvis.ico2s.org/dev/seednet/#/static/Data) which is a co-expression network that has 8,621 nodes with 502,173 interactions. The nodes were pre-clustered with MCODE into 136 clusters. It took AutoAnnotate a few minutes to annotate the clusters (on a MBP 2012), which is acceptable, and then about 10 seconds to generate a summary network. The produced cluster labels were  based on the annotation of the clustered genes. The labels were not very informative because there were hardly any common words in the annotations. This was obvious from the words in the labels, and from the output of the WordCloud app for a cluster. For a future version of the app it might be an idea to scale the font of the label by the frequency of the chosen words (now the font can be scaled by the cluster size), to indicate how descriptive a label is for a certain cluster.

 The manuscript is well written and to the point.

 In the following two lines the plural “they” refers to the singular “user”:

 “The USER may create as many Annotation Sets as THEY like, and can easily switch between them.”

 “if not available the USER is presented with a web link to the App Store page for clusterMaker2 from

 which THEY can install it.”

 AutoAnnotate is a useful addition to the collection of Cytoscape apps, and will undoubtedly help many users to create an informative summary of a complex network.

I have read this submission. I believe that I have an appropriate level of expertise to confirm that it is of an acceptable scientific standard.

# Peer review: Referee response for version 1

**DOI:** 10.5256/f1000research.9783.r16447

**Published:** 2016-10-04

**Authors:** Pablo Porras Millán

**Affiliations:** 1 European Molecular Biology Laboratory, European Bioinformatics Institute, Hinxton, UK

The authors present a new Cytoscape app, AutoAnnotate, that integrates topological cluster search with automatic annotation in order to help visualize underlying structures in complex networks. The tool comes to complement previous work by the same authors in other Cytoscape apps such as EnrichmentMap or WordCloud. This app provides a much-needed tool for semi-automatic annotation and visualization of complex network data.

 I found the app particularly useful when used to visually annotate and summarize term enrichment analysis results, which was its original design motivation. Used in combination with EnrichmentMap it provides users with enhanced visualization options on popular tools, such as BiNGO, g:Profiler or DAVID.

 The manuscript is clearly and concisely written, providing a nice introduction on the app functionality, its motivation and how to start using it. The tutorial referenced in the text is also quite useful, I have followed it to test the app with different use cases and I could get satisfactory displays without spending much time tweaking my results. The examples shown were also relevant and helped showing the app capabilities, although I would have welcomed an example using third party tool data, such as sorting out the network visualization output of BiNGO.

 To summarize, this is a valuable tool that I hope to see much used in network annotation visualization in the future.

I have read this submission. I believe that I have an appropriate level of expertise to confirm that it is of an acceptable scientific standard.
